# Does Living in a Fatherless Household Compromise Educational Success? A Comparative Study of Cognitive and Non-cognitive Skills

**DOI:** 10.1007/s10680-017-9414-8

**Published:** 2017-03-23

**Authors:** Jonas Radl, Leire Salazar, Héctor Cebolla-Boado

**Affiliations:** 1 Department of Social Sciences, Carlos III University of Madrid, Madrid, Spain; 2 Department of Social Stratification, UNED, Madrid, Spain

**Keywords:** Household structure, Education, Locus of control, Father absence, International comparison, Numeracy

## Abstract

This study addresses the relationship between various family forms and the level of cognitive and non-cognitive skills among 15- to 16-year-old students. We measure cognitive skills using standardized scores in mathematics; non-cognitive abilities are captured by a composite measure of internal locus of control related to mathematics. A particular focus lies on father absence although we also examine the role played by co-residence with siblings and grandparents. We use cross-nationally comparable data on students participating in the Programme for International Student Assessment’s release for 2012. By mapping inequalities by family forms across 33 developed countries, this study provides robust cross-country comparable evidence on the relationship of household structure with both cognitive and non-cognitive skills. The study produces three key results: first, the absence of fathers from the household as well as co-residence with grandparents is associated with adverse outcomes for children in virtually all developed countries. Second, this is generally true in terms of both cognitive and non-cognitive skills, although the disadvantage connected to both family forms is notably stronger in the former than in the latter domain. Finally, there is marked cross-national diversity in the effects associated with the presence in the household of siblings and especially grandparents which furthermore differs across the two outcomes considered.

## Introduction

The sociology of education has studied extensively how inequalities by social origin are reflected in indicators of students’ attainment and performance (Breen and Jonsson 2005), showing consistently that household resources and family structure are among the most important predictors. The implications of parental separation or divorce for children’s well-being are of special interest here (Amato 2010), and the recent upsurge in the numbers of affected children has led some scholars to suggest an increasing differentiation of social destinies (McLanahan 2004). Even though findings are sensitive to the methods used and to the ability to address endogeneity and selection issues (Kim 2011), a tentative consensus has emerged about an actual causal penalty existing on a broad set of outcomes. Children experiencing a divorce or a parental separation are more prone to suffer more externalizing behaviour, to show lower mental well-being than those living with two parents (Dronkers 1999; Gähler and Palmtag 2015) and, more generally, to experience poorer health (Amato and James 2010; Chase-Lansdale et al. 1995; Uphold-Carrier and Utz 2012). They are also more likely to experience crucial life transitions at earlier ages, such as leaving the parental home, entering a union and becoming a parent (Ní Bhrolcháin 2001), and to break their unions themselves (Dronkers and Härkönen 2008). Most importantly for the present study, children from non-intact families exhibit worse educational outcomes (McLanahan et al. 2013), whether measured by scores or grades (see Erman and Härkönen 2017) or by characteristics of the educational trajectory such as the type of track chosen, grade retention and final attainment (Bernardi and Boertien 2016a).

In line with this growing interest in the effect of family structure and household configuration on children’s development, this study examines the role of specific family forms for two outcomes—numeracy and locus of control—that correlate with long-term educational achievement and jointly capture both cognitive and non-cognitive skills. We have three research questions. First, the literature has shown, for different countries, a significant penalty associated with the absence of fathers from the household. While most studies are concerned with one particular event causing father absence, namely parental divorce, we broaden the scope and ask to what extent the disadvantage associated with this family form in general is an international regularity, drawing on data from a large number of countries. Second, we ask whether co-residence with grandparents or siblings influences our two outcome measures and the extent to which there are any international patterns in the associated (dis)advantages. Our third research question taps further into the underlying processes addressing the role of co-residing grandparents or siblings in potentially offsetting the absence of the father in the household. We specifically assess whether the presence of these members of the extended family correlates differently with cognitive and non-cognitive skills, depending on the presence or absence of the father. To answer these questions, we exploit cross-national evidence from 33 member countries of the Organisation for Economic Cooperation and Development (OECD). Specifically, we use data from Programme for International Student Assessment (PISA) 2012, which allows us to diversify the standard approach of concentrating exclusively on cognitive outcomes. This data includes information on both standardized test scores and students’ beliefs that putting effort into their school tasks enhances their educational performance in mathematics.

Our paper contributes to the literature in three significant ways. First, we address the processes behind inequalities by systematically comparing two different dependent variables (mathematics scores and locus of control), capturing both cognitive and non-cognitive skills. To the best of our knowledge, there exists no scholarly contribution addressing international regularities in non-cognitive outcomes, nor comparing cognitive and non-cognitive measures. Second, we analyse the extent to which different family configurations (the absence of fathers as well as the presence of grandparents and siblings) are associated with disadvantages in mathematics scores and locus of control, and investigate whether offsetting processes are at work. Third, we explore inequalities by family structures internationally, mapping disparities across developed countries. In summary, our paper addresses different research strands, including the literature on the effects of family forms on socio-economic attainment and the debates around the role of cognitive versus non-cognitive skills for educational success.

Because of data limitations, we do not consider the different circumstances leading to the father being absent from the household (death, divorce or separation, the couple living apart together, etc.) (cf. Biblarz and Gottainer 2000). Nor can we identify the exact mechanisms (socio-economic resources, parental involvement and support, conflict and stress, etc.) explaining the potential penalty incurred by father absence (e.g. Sigle-Rushton et al. 2014). In contrast to the comparative literature testing hypotheses at the meso- and macro-level (see below), we do not aim to explain cross-country variation in outcomes but rather at mapping the potential penalties in the cognitive and non-cognitive domains across a large number of countries.

## Cognitive and Non-cognitive Skills

Traditionally, social scientists have used cognitive and behavioural outcomes as central indicators to measure (socio-economic, ethnic, gender or other) group differentials in education. In order to quantify inequalities by family form, the largest share of this literature analyses cognitive outcomes such as literacy and numeracy derived from test scores, and how they influence outcomes such as transition rates among students with different characteristics from compulsory to non-compulsory education, the choice of an academic versus a vocational track in the educational system or the attainment of a particular educational diploma (McLanahan et al. 2013). The traditional way of measuring cognitive skills in sociology has been in the form of school results such as grades or examination scores (Boudon 1974). More recently, there is growing use of standardized measures of cognitive skills above and beyond schooling, already starting in the early stages of educational careers (Sullivan et al. 2013). Increasing attention is paid to differences between educational systems (Hanushek and Woessmann 2012). Over the last two decades, different organizations have developed efforts to validate instruments measuring competences and skills internationally (for example, the “Key Competences” as defined by the European Commission, or the “Life skills” by UNESCO). This trend is reflected in the standard test scores provided by the benchmark international studies of student performance conducted by the Organisation for Economic Cooperation and Development (OECD) or the International Association for the Evaluation of Educational Achievement (IEA). Yet “achievement-related skills” (O’Connell and Sheikh 2008) and outcomes are far from being strictly cognitive. Indeed, research has recently incorporated a new focus on non-cognitive outcomes (Heckman and Kautz 2012), which are arguably equally important in the process of learning. Scores in standardized tests are no longer univocally considered the single strongest predictor of attainment rates (Zau and Betts 2008). Instead, scholars have argued that long-term educational success depends critically on “soft” factors such as self-control, discipline and conscientiousness (Duckworth and Seligman 2005).

The concept of “non-cognitive skills” is blatantly broad and covers a wide array of phenomena that have been discussed using varying labels in different disciplines including self-efficacy, motivation, perseverance, self-control, social competence, resilience, coping and creativity (Gutman and Schoon 2013). Some scholars approach non-cognitive skills through a motivational component that refers to the level of aspiration and ambition. Persistence, perseverance or “grit” (Duckworth et al. 2007) refer to self-control and the discipline to exert effort over extended time. The famous Stanford Marshmallow experiment carried out in the late 1960s was among the first studies to highlight the importance of non-cognitive skills, in this case the ability to delay gratification (Castillo et al. 2011). Non-cognitive skills further include interpersonal skills such as empathy and sociability, self-esteem, self-confidence or emotional stability and maturity. In this paper, we specifically look at locus of control (Antunes and Ahlin 2014), which is known to be a significant determinant of pro-social behaviour (Meier et al. 2008) and educational success (Au 2015). Such personality characteristics are typically assumed to be stable across the life course (Fraley and Roberts 2005; Cobb-Clark and Schurer 2013) as opposed to purely cognitive skills which are known to be less stable over time (Cooper et al. 1996; Tiruchittampalam et al. 2016). Based on this notion, one could expect a more limited influence of changes in family forms on non-cognitive skills as compared to cognitive skills.

Research in the educational field has analysed the extent to which non-cognitive skills and actual performance correlate. If results in mathematics are examined, the evidence shows a clear negative association between anxiety towards this subject and aptitude and achievement across all grade levels (OECD 2015a). This finding has been reported consistently for students in secondary school, but, interestingly, similar results hold in the first grades of primary school (Wu 2014). Analogously, internal locus of control is positively associated with performance: students in the USA and Japan who attributed school success in mathematics to factors that they could control, such as effort, achieved better mathematics test scores than students who tended to give more weight to external factors such as luck (House 2006). Last, the positive correlation between self-efficacy and different indicators of mathematics performance has been widely shown (OECD 2015b). Beechum (2012) presents an extensive review of the literature covering real interventions developed to change non-cognitive skills, demonstrating that scholarship is far from reaching a consensus regarding the malleability of these outcomes.

Together with a traditional measure of cognitive performance such as numeracy, we look at “locus of control”, which is considered a key non-cognitive skill. Locus of control is the belief that life events are causally attributable to one’s own actions, and has been used extensively since the 1980s to explain differences in effort, especially among children. According to this approach, a high degree of external locus of control results in the belief that fate or luck is the responsible factor for what happens, as opposed to a high degree of internal locus of control, when someone believes that the driving force of success is ability and effort exerted in one’s actions (Rotter 1975). Experiments proved that the lack of control on one’s life created, in the long run, several psychological problems including depression (Garber and Seligman 1980).

## Family Forms and Educational Success

### The Influence of Father Absence

There is ample evidence on family arrangements involving the absence of one of the parents, usually the father, being negatively correlated with educational success of students (McLanahan et al. 2013). Scholars have analysed the detrimental impact on a variety of outcomes such as test scores (Cherlin et al. 1991), grades (Grätz 2015), grade retention (Pong and Ju 2000), track selection (Jonsson and Gähler 1997), and attitudes about school and educational aspirations (Astone and McLanahan 1991). When focusing on the final level of educational achievement and/or years of schooling attained, the negative influence exerted by father absence seems to be especially pronounced. Although this penalty is particularly marked in the USA, where the evidence is more abundant, the evidence from other national settings goes in a similar direction (Keith and Finlay 1988; Ermisch and Francesconi 2001; Björklund and Sundström 2006; Bernardi and Radl 2014; Bernardi and Boertien 2016a, b).

Much of the literature focusing on this penalty deals with the search for the “true” (i.e. causal) effect of father absence (most often, in the literature, stemming from separation or divorce). Scholars have used increasingly more complex empirical strategies trying to account for selection bias and endogeneity (see also the Introduction to this Special Issue) and, correspondingly, research results vary a great deal depending on the techniques used and the type of data available in different national settings. All in all, estimates of the causal effect tend to be significantly more limited in size, sometimes even negligible, compared to those obtained using simpler strategies.

Important differences by family types have also been reported when examining the non-cognitive domain. Most research available focuses on the USA and the UK and has shown an impact of father absence on different indicators of psychological distress and emotional problems (Cherlin et al. 1998; Ermisch et al. 2004), locus of control and self-esteem (Sun and Li 2002), externalizing behaviour and problems with peers (Cavanagh and Huston 2008). Many contributions addressing non-cognitive education-related outcomes have also concentrated on the effects of divorce or separation, with mixed results depending on the outcome considered, the sample used and the timing of couple disruption. When locus of control—our main non-cognitive trait of interest in this paper—is examined, interesting results emerge. Kim et al. (1997) analyse the role of locus of control as a moderator and mediator of stress in children between 8 and 12 years of age whose parents had divorced during the previous two years. On the one hand, they find that locus of control moderated the impact of stress on psychological symptoms. More specifically, having a causal understanding of why positive events occur helped children cope with divorce-related stress, which the authors interpreted as happening by virtue of a “sense of secondary control” in the presence of uncontrollable stressors. On the other hand, the study found evidence for locus of control acting as a mediator, albeit only to a limited extent. Supposedly, the experience of a stressful, exogenous shock such as parental divorce leads to loss of control beliefs among children, which consequently boosts undesired psychological symptoms.

Although studies analysing school success of children living in different family configurations have mostly focused on a single country, some scholars have speculated theoretically about the influence of family structure on a student’s outcomes in different settings: on the one hand, it has been suggested that the role of family configurations should be weaker in richer countries where other public resources can substitute family stimuli (Chiu 2010); on the other hand, the opposite prediction has been made based on the higher involvement of parents in richer nations (Sandberg and Hofferth 2001). Yet a third view (Scott et al. 2013) suggests that family structure may have a lower impact on educational outcomes in low-income countries because of the dominant role of other structural obstacles to educational attainment (e.g. health, nutrition, quality of education, seasonal labour demands) that overshadow the possible influence of family structure. Empirically, studies adopting a multicountry perspective have confirmed a negative correlation between single parenthood in both literacy (Hampden-Thompson 2013) and mathematics scores (de Lange et al. 2014) and have analysed variables, such as the varying availability of certain family policies and the differing prevalence of single-parent children across schools. The link with long-term educational attainment has also been demonstrated (Bernardi and Boertien 2016b). Of course, it is complicated to explain cross-national differences in the impact of single parenthood on education since its meaning differs across cultural settings (Park 2007), and its implications can also vary as a function of the institutional design of educational systems (Bernardi and Radl 2014).

### The Presence of Siblings and Educational Outcomes

Research indicates that having siblings is negatively associated with a student’s cognitive ability (Steelman et al. 2002; Lawson et al. 2013) and final educational attainment (Sandefur et al. 2006; Kalmijn and van de Werfhorst 2016) is abundant and has most often been framed within the resource dilution approach. It originates in the classic idea of a trade-off between the quantity and quality of children (Becker and Lewis 1973). In a nutshell, this approach predicts that, since family resources are finite, additional children in the household reduce the amount of resources that parents can allocate to each child (Downey 2001). Other hypotheses such as the less stimulating intellectual environment that characterizes, according to the confluence model, larger families, similarly predict negative effects of sibship size on children’s education. In contexts such as the USA in which access to university entails a very significant financial burden to families, the validity of the dilution approach has been consistently confirmed as regards educational attainment: families with a larger sibship face more difficulties in funding all of their children’s college attendance. When university is state-funded and/or attendance costs are less strenuous, attention should focus less on attainment and more on performance. Part of the negative effect that is most often found might be due to processes associated with, but theoretically distinct from, sibship size such as birth order (see Härkönen 2014), sex of the siblings and even the extent to which parents compensate or reinforce initial deficits and capabilities of their offspring (Hsin 2012; Bernardi 2014).

The study of non-cognitive outcomes, significantly less developed in the literature, reveals a completely different pattern. Even though parental attention and involvement need to be shared as family size increases, siblings themselves might constitute an independent source of stimuli and emotional support. Evidence from large-scale studies suggests that growing up with at least one sibling is associated with better social and interpersonal skills, more self-control and less externalizing problems, although all these benefits tend to disappear when sibship size is three or larger (Downey and Condron 2004). Although the literature on these non-cognitive outcomes is scarce, birth order or sex of the siblings is likely to play a role as well. Birth order seems to be a mediator on the relationship between sibship size and non-cognitive outcomes. For instance, having older siblings appears to be associated with better mental health scores, while having younger brothers or sisters is related to poorer performance in this domain (Lawson and Mace 2010).

### The Role of Co-residence with the Grandparents

In the last number of years, research on the role of the extended family, and particularly grandparents, has become more common. Scholars have tried to assess their role to explain social mobility and status attainment processes and, to a more limited extent, schooling. Specifically, the influence of extended family on various types of outcomes such as academic achievement (Falbo 1991) and cognitive development (Modin and Fritzell 2009) has been addressed. Recent research incorporating the grandparents’ generation when explaining children’s school outcomes has produced mixed results. Jæger (2012) found that grandparents’ socio-economic characteristics in the USA affect children’s schooling only when the parents have limited resources themselves, a finding that has been interpreted as evidence of the existence of compensatory mechanisms across generations. Yet, in the Netherlands, Bol and Kalmijn (2016) found no significant direct influence of different types of grandparental resources on children’s educational attainment. In Denmark, grandparents’ cultural, rather than material, capital is associated with children’s choice between an academic and a vocational track (Møllegaard and Jæger 2015).

Whereas research on the impact of transfers from non-resident grandparents described above is developing fast, the implications of co-residence with the extended family have received less attention. Kreidl and Hubatková (2014) found that living with grandparents and having a large sibship are both associated with lower reading scores among secondary students. The interaction between the two is, moreover, negative, suggesting that co-residence of multiple generations is not able to moderate the adverse impact of having more siblings on cognitive outcomes. In addition, this pattern becomes more evident in more developed countries, suggesting some selection effect by which co-residence of multiple generations tends to take place among families with fewer resources. Children co-residing with grandparents may thus exhibit relatively worse cognitive outcomes in countries where this family form is more uncommon. This selection effect has been similarly found when analysing other outcomes such as the economic position of households of single mothers co-residing with grandparents in Asia (Shirahase and Raymo 2014). More generally, some scholars have interpreted that contexts in which the association between sibship size and education is weak, tend to be those with social norms promoting large families and, specifically, a strong involvement of the extended family and multiple generations in childrearing (Downey 2001). Analyses of rural China have for instance shown that in living arrangements that include three generations, when grandparents are highly educated, they tend to mitigate the likelihood of school-age children dropping out of school (Zeng and Xie 2014).

### Summary of Expectations

Drawing on previous findings and on the available theoretical contributions, we empirically assess the following expectations. First, we expect father absence to be systematically associated with lower cognitive and non-cognitive outcomes relative to families with two resident parents. The absence of one of the parents, and specifically the father, tends to go along with fewer resources of all kinds (material, cultural, emotional and social) in the household, and this scarcity has adverse implications for school success. Second, in line with both the resource dilution and the confluence model hypotheses, we expect the presence of siblings to be detrimental to cognitive outcomes, under *ceteris paribus* conditions. On the contrary, we expect siblings to supply certain emotional support to their brothers and sisters that may enhance their non-cognitive outcomes. Third, we expect co-residence with the grandparents to be associated with poorer educational performance, both in terms of cognitive and non-cognitive characteristics. Even though our data do not allow for investigating the causal mechanisms at work, we argue that selection into this type of family configuration could explain this negative association. Lastly, we explore the possibility that the presence of siblings and grandparents could, to some extent, compensate the penalty associated with father absence. This expectation seems plausible especially for non-cognitive skills, which are arguably more susceptible to personal and emotional support and less responsive to adverse life events, such as parental divorce or death, that are frequent triggers of non-standard family forms.

## Data and Variables

The comparative analysis on the impact of household configurations and, more specifically, on the impact of father absence on educational success is hindered by the lack of available data. Our analyses draw on the 2012 edition of PISA, an international survey that assesses the competencies of 15- to 16-year-olds in reading, mathematics and science (the 2012 round had a focus on mathematics). One of its advantages is the large sample size, which provides a sufficient number of observations corresponding to rather uncommon family forms such as children living in fatherless households who, in some cases, would live with their siblings or grandparents. So as to avoid excessive international heterogeneity, we restrict our analysis to member countries of the OECD. Appendix includes a list of the 33 countries included and their respective sample size (Appendix Table 5).

Table 1 summarizes the variables used in the analysis. We examine two dependent variables. The first one is numeracy test scores, which we use as a measure of cognitive outcomes. The second one is locus of control, our selected indicator for non-cognitive outcomes.

**Table 1:** Descriptive statistics

	Mean	SD	Min	Max
Continuous variables				
Numeracy (maths test score)	0.00	1.00	−4.10	4.28
Locus of control (internal)	0.00	1.00	−4.56	4.38
Mother’s years of education	12.68	3.25	1.27	22.26
Age in years	15.78	0.29	15.11	16.41

	Proportion (%)
Dichotomous variables	
Father absent from household	11.72
Grandparents in the household	15.01
Siblings in the household	86.60
Female	50.13
Native born	89.33

*Source* PISA 2012, 33 countries, *N* = 259,652

*Note* Rubin’s rules applied to account for cross-imputation variation

Numeracy is the result of the test in mathematics taken by all students.^1^ Locus of control is a continuous scale where higher values represent more internal locus of control. This variable was built by merging different questions included in the student questionnaire on the general topic of mathematics learning. These variables ask about the degree of agreement with the following statements, all referring to mathematics: (1) one can succeed with enough effort; (2) doing well is completely up to me; (3) if I wanted, I could perform well; and (4) I perform poorly regardless of the effort I put in. Each of these questions provides four possible answers: strongly agree, agree, disagree and strongly disagree. Exploratory factorial analysis was conducted to synthesize the four items into a single continuous score. Only one score was retained using the criterion eigenvalue >1 (1.433). In the appendix the factor loadings are shown in detail (Table 6). To make the scales pertaining to numeracy and locus of control comparable, both have been standardized (mean = 0; SD = 1).

Household structure is measured using three dummy variables. The first one registers whether the father co-resides with the children (1) or not (0). Note that because of data constraints, we only know about the presence of the father in the household, but we ignore the reason for his absence. Moreover, we are unable to distinguish between biological- and stepfathers. The presence of grandparents (grandfather, grandmother or both) and siblings is likewise registered using dichotomous variables. The data register only the presence of any siblings in the household, not the number of siblings that students have. Table 5 (Appendix) shows the prevalence of each family form in the 33 countries.

We control for the mother’s education, expressed in years corresponding to the International Standard Classification of Education (ISCED) (for details, see OECD 2014: 444). Because of the prominence of the selection argument in the divorce literature, it is standard procedure by now to control for family background when estimating the effect of parental separation on children’s well-being.^2^ As for the students’ characteristics, we control for their age, sex and migrant status (1 being native born and 0 a student born in a different country from the one in which he/she takes the test).

We used multiple imputation by chained equations to account for missing data. Ten sets of imputations were used. In addition to the variables included in the data analysis, we incorporated the following variables in the imputation procedure. First, we added a synthetic index of economic, social and cultural status (ESCS, provided by PISA) to the equation. This composite measure contains the highest level of education of either the father or the mother (in number of years according to the ISCED classification), the highest occupational status (ISEI) between the two parents and the number of home possessions. As set out above, we decided not to include these contemporary measures of socio-economic well-being in the explanatory data analysis, but used ESCS in the imputation procedure as an additional source of information to account for missing data. Second, given the nature of our outcome variables, father’s education was also used as a separate variable in the imputation procedure. Third, the imputation procedure also distinguished between brothers and sisters in the household, but we only report the presence of siblings in general as no systematic gender differences in the effects of siblings on education were found.

## Method

Given the linear form of our dependent variable and the structure of our data (students in different countries), we use hierarchical linear models (HLM) with random effects. From a theoretical point of view, we are not interested in testing the validity of explanations at the macro (country)-level, nor in the way country characteristics interact with predictors at the individual level (cross-level interactions). The random intercept multilevel model decomposes the residual in two random terms, one for the individual (*ε*_*ij*_) and one for the aggregate level.

$$y_{ij} = \gamma_{00} + u_{0j} + \beta_{1} x_{1} + \cdots + \beta_{n} x_{n} + \varepsilon_{ij}$$

where *γ*_00_ is the average intercept of all countries considered, while *u*_0*j*_ is a random term for the specific average intercept of each country. This second random term can be considered as a sort of latent variable capturing the specificity of each cluster that can eventually be explained modelling the variation existing within and across clusters under a full model specification. The decomposition of the regression error into *u*_0*j*_ and *ε*_*ij*_ allows for a proper quantification of the effect of the clustering of individual observations and a reliable estimation of the effect of level 1 and level 2 independent variables.

As is conventionally done when applying HLM models to a limited set of countries, we estimate our models using restricted maximum likelihood, which takes into account the number of fixed-effects parameters estimated and the remaining degrees of freedom, before moving to the estimation of the variance of random components.

## Results

Table 2 displays our first findings for both numeracy and locus of control. We use a parsimonious model specification so as to plainly control for basic socio-demographics (students’ sex, age, social background as measured by the mother’s level of education and migrant status).

**Table 2:** Random-slopes hierarchical models

	Numeracy (maths test score)		Locus of control (internal)	
	*b*	SE	*b*	SE
Constant effects				
Father absent from household	−0.143***	0.014	−0.059***	0.012
Grandparents in the household	−0.180***	0.006	−0.034***	0.008
Siblings in the household	−0.046***	0.005	0.025***	0.006
Female	−0.136***	0.004	−0.152***	0.004
Age in years	0.166***	0.006	−0.017*	0.008
Native born	0.299***	0.006	−0.108***	0.007
Mother’s years of education	0.070***	0.001	0.017***	0.001
Constant	−3.503***	0.107	0.178	0.141

	Var	SE	Var	SE
Random effects				
Father absent from household	0.005	0.002	0.002	0.001
Constant	0.062	0.015	0.045	0.011
Covariance	−0.007	0.004	0.000	0.003
Individual residual	0.791	0.002	0.944	0.003

*Source* PISA 2012, 33 countries, *N* = 259,652

Rubin’s rules applied to account for multiple imputation (10 imputations)

*p* values: + 0.1, * 0.05, ** 0.01, *** 0.001

It is evident from these estimates that having an absent father yields a negative effect, for numeracy as well as locus of control (−0.143 and −0.059, respectively): in our sample of OECD countries, students in fatherless households score a lower average in mathematics and are more prone to attribute their educational success to external factors than those in two-parent families. Since the scale of both dependent variables is standardized, it is straightforward to see that the “penalty” for not living with the father is markedly larger for numeracy test scores (the coefficient corresponds to 14 original PISA scale points less before standardization) than for locus of control.

**Fig. 1:**
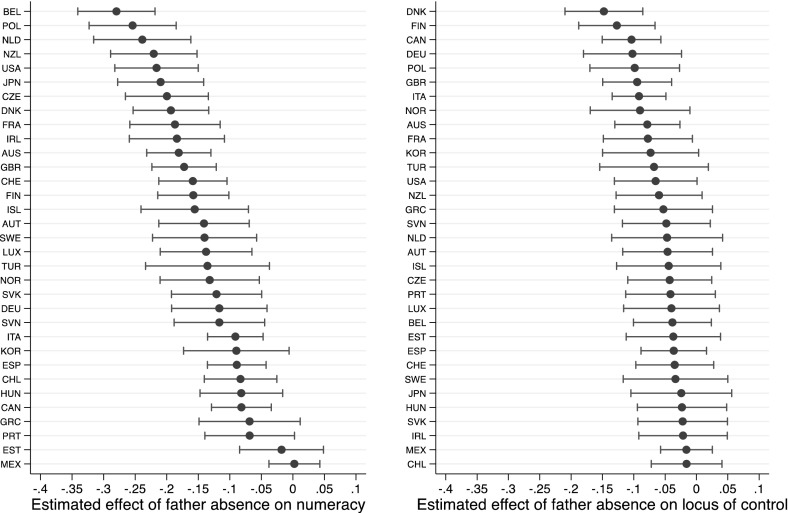
Between-country variation in the estimated effect of father absence on cognitive and non-cognitive outcomes. *Note* Models control for gender, age, foreign born, mother’s education as well as presence of grandparents and siblings in the household

Looking at the random component of the model, in Fig. 1 we turn to explore the between-country variation in the parameter corresponding to father absence. The figure plots the sum of the average effect of father absence shown in Table 2 plus the random deviations from it; thus, each dot describes the total country-specific penalty associated with father absence. The left-hand panel of the figure corresponds to the country-specific penalties associated with father absence in numeracy, and the right-hand one to penalties associated with locus of control. In sum, the absence of the father is unanimously disadvantageous for numeracy in virtually all settings; even though the size of the estimated effects differs internationally, it is significantly negative for mathematics scores almost everywhere (except in Mexico, Estonia, Portugal and Greece where it does not significantly differ from zero). By contrast, we find that the estimates for locus of control indicator are markedly smaller, never higher than a seventh part of a standard deviation, and in most countries not significantly different from zero.

To provide additional background information on the variation at the country level and explore the possible role of selection effects, Table 7 in the appendix shows pairwise cross-national correlations between the estimated coefficients and the prevalence of the different family forms. In the case of father absence, it can be noted that the estimated penalties for both outcomes are positively correlated at the country level (*r* = 0.22). The correlations between the prevalence of father absence across our sample of countries and the estimates obtained for numeracy and locus of control are very low (*r* = −0.09 and 0.04, respectively). There appears to be no particular pattern, therefore, in the size of the penalties related to how common households with an absent father are in a particular setting.

The models presented in Table 2 furthermore show that students living with at least one of their grandparents tend to score lower in both numeracy and internal locus of control (−0.180 and −0.034, respectively). These results suggest that this type of family configuration could be regarded as a source of disadvantage for educational outcomes although, of course, the mechanisms explaining this finding are difficult to detect using cross-sectional data. It is likely that selection effects into different family arrangements are at work among children with prior performance or attitudinal issues or children from deprived or otherwise needy families.

As for co-residence with siblings, the longstanding idea, manifest in the resource dilution hypothesis explained above, that having a larger sibship is detrimental for educational outcomes, is generally sustained here for numeracy despite the small magnitude of the estimated effect (−0.046). Yet, interestingly, the result does not hold for locus of control. Rather, co-residence with siblings appears to provide children with some sort of emotional support that boosts self-confidence and improves internal locus of control, although this is again a small effect (0.025).

Results for the control variables are in line with the literature: as expected, girls have lower outcomes in mathematics compared to boys, children of families with more resources (in this case, mother’s years of education) rate better than their counterparts in less advantaged households, and native students score higher in cognitive tests but tend to show lower internal locus of control than students with an immigrant origin, something that is in line with the well-known immigrant optimism hypothesis (Kao and Tienda 1998).

**Fig. 2:**
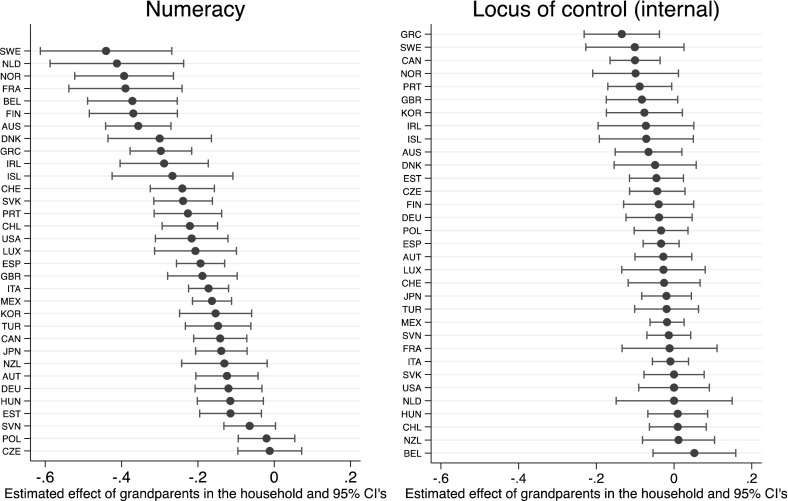
Between-country variation in the estimated effect of co-residence with grandparents on cognitive and non-cognitive outcomes. *Note* Models control for gender, age, foreign born, mother’s education as well as presence of fathers and siblings in the household

Figure 2 shows results from multilevel models with the same specification as in Table 2, but in this case, the random slope refers to co-residence with grandparents. As before, Fig. 2 displays the sum of the fixed and random effects.

Living with grandparents is systematically associated with adverse results for children’s mathematics scores (panel on the left). Moreover, in a significant share of the countries, the reduction in scores is relatively large, and the estimate is significant in all but three countries (Czech Republic, Poland and Slovenia). In the majority of countries, the presence of at least one grandparent is also harmful in the case of locus of control, although the penalty is markedly smaller than for test scores. Children in this type of family arrangement tend to score lower in the internal locus of control scale, with the exception of only four countries—New Zealand, Chile, Hungary and Belgium—where the estimate is positive, albeit close to zero. Overall, the estimated effects are less heterogeneous across countries than for absent fathers (the two penalties are mildly correlated, *r* = 0.17). As Table 7 in the appendix shows, the correlation between how common co-residence with grandparents is in our 33 countries and the size of the estimates for the cognitive measure is moderate and positive (*r* = 0.66), while it is positive but weak in the case of our non-cognitive outcome (*r* = 0.11).

**Fig. 3:**
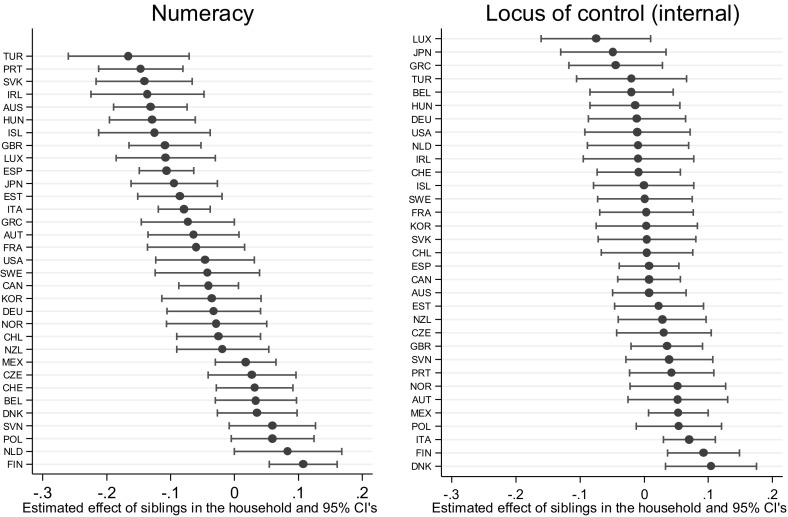
Between-country variation in the estimated effect of co-residence with siblings on cognitive outcomes and non-cognitive outcomes. *Note* Models control for gender, age, foreign born, mother’s education as well as presence of fathers and grandparents in the household

In Fig. 3, we analogously show the country-specific markers representing the estimates associated with the presence of siblings in the household. For either outcome, there is considerable variation in this effect across countries, and identifying systematic patterns in the results is not straightforward. Given the varying sign of the coefficient across countries, the results do not provide robust international support for neither the resource dilution hypothesis nor the idea of siblings boosting non-cognitive skills. Nevertheless, there is a majority of countries where the coefficient is negative for numeracy, whereas the effect of siblings on locus of control is positive, though close to zero, in all countries except Denmark, Finland, Italy and Mexico. In line with the results shown in Figs. 1 and 2, the magnitude of the estimates is again larger overall for test scores than for locus of control. While this evidence should be interpreted with caution, the consistency of this finding reinforces the idea that non-cognitive outcomes tend to be less responsive or more resilient to life events than cognitive skills. According to this interpretation, the detrimental influence on students’ educational success by some family arrangements appears to be operating through dwindling performance rather than the undermining of confidence in effort as a means to achieve goals. However, the estimates of the country-specific penalties for both outcomes correlate strongly (*r* = 0.41). The correlation between the prevalence of siblings’ co-residence across countries and our estimate for numeracy scores is negative and weak (*r* = −0.21). The correlation is also negative but much stronger (*r* = −0.42) when we look at locus of control instead (Table 7), suggesting that siblings’ presence may only be helpful in contexts where children co-reside with both parents.

In Table 3, we show the results of several random–constant hierarchical models. For each outcome, we present a set of specifications with the main effects pertaining to the family forms that we have discussed above and the same set of control variables, but now we also include interaction terms between father absence and co-residence with grandparents (first and second columns in the panel for each outcome) and siblings (third and fourth columns), respectively. In columns 5 and 6, they are introduced jointly. This analysis aims to elucidate whether the disadvantage entailed by co-residence with grandparents holds in households where the father is absent compared with those with both parents present. In addition, it examines how the presence of siblings is associated with educational results when we distinguish between two-parent families and absent-father households.

**Table 3:** Random–constant hierarchical models with interaction terms

	Numeracy (maths test score)						Locus of control (internal)					
	*b*	SE	*b*	SE	*b*	SE	*b*	SE	*b*	SE	*b*	SE
Constant effects												
Father absent from household	−0.138***	0.006	−0.150***	0.011	−0.165***	0.011	−0.069***	0.007	−0.068***	0.014	−0.077***	0.014
Grandparents in the household	−0.191***	0.006	−0.179***	0.006	−0.190***	0.006	−0.040***	0.008	−0.033***	0.008	−0.040***	0.008
Father absent × grand parents (hh)	0.095***	0.016	–	–	0.096***	0.016	0.057**	0.018	–	–	0.057**	0.018
Siblings in the household	−0.046***	0.005	−0.054***	0.006	−0.054***	0.006	0.026***	0.006	0.023**	0.007	0.023**	0.007
Father absent × siblings (hh)	–	–	0.035**	0.013	0.036**	0.013	–	–	0.010	0.016	0.011	0.016
Female	−0.136***	0.004	−0.136***	0.004	−0.136***	0.004	−0.152***	0.004	−0.152***	0.004	−0.152***	0.004
Age in years	0.166***	0.006	0.166***	0.006	0.166***	0.006	−0.017*	0.008	−0.017*	0.008	−0.017*	0.008
Native born	0.299***	0.006	0.299***	0.006	0.299***	0.006	−0.108***	0.007	−0.108***	0.007	−0.108***	0.007
Mother’s years of Education	0.070***	0.001	0.070***	0.001	0.070***	0.001	0.017***	0.001	0.017***	0.001	0.017***	0.001
Constant	3.505***	0.106	3.500***	0.106	3.497***	0.107	0.178	0.141	0.179	0.141	0.181	0.141

	Var	SE	Var	SE	Var	SE	Var	SE	Var	SE	Var	SE
Random effects												
Constant	0.060	0.015	0.060	0.015	0.060	0.015	0.045	0.011	0.046	0.011	0.045	0.011
Residual	0.791	0.002	0.791	0.002	0.791	0.002	0.944	0.003	0.944	0.003	0.944	0.003

*Source* PISA 2012, 33 countries, *N* = 259,652

*Note* Rubin’s rules applied to account for multiple imputation (10 imputations)

The interaction between father absence and living with at least one grandparent is positive in the two outcomes considered here. However, for numeracy, the magnitude of the interaction term is generally smaller than either of the main effects. Co-residence of grandparents in households in which the father is not present apparently cannot fully compensate for the original disadvantage associated with these household arrangements. In fact, as Table 4 illustrates (showing the calculations of the estimated main and interactive effects for each family configuration using the models shown in Table 3), the family form that is associated with the poorest results in numeracy appears to be households with fathers absent and grandparents present.

**Table 4:** Estimated family form effects

	Numeracy		Locus of control	
	Co-residing grandparents		Co-residing grandparents	
	Yes	No	Yes	No
Estimated difference vis-à-vis two-parent child without co-residing grandparents or siblings				
Father absent				
Siblings present	−0.277	−0.183	−0.026	−0.043
Siblings absent	−0.259	−0.165	−0.060	−0.077
Father present				
Siblings present	−0.244	−0.054	−0.017	0.023
Siblings absent	−0.190	Ref.	−0.040	Ref.

*Source* PISA 2012, authors’ calculations based on full models shown in Table 3

Looking at it from the other way, single-mother households (without grandparents present) seem to be similarly negatively selected as are multigenerational households with both parents and grandparents. Our results thus do not support the idea that, across OECD countries on average, co-residence would imply an improvement in test scores. However, our analysis indicates that the presence of grandparents may partly compensate the disadvantage associated with father absence when it comes to locus of control. In any event, more elaborate research designs involving longitudinal data are necessary to test these hypotheses in a causal fashion.

Interacting father absence with the presence of siblings in the household reveals a more inconsistent pattern. As regards numeracy, the main effects of both father absence and co-residence with brothers or sisters are negative. Even though the interaction term is positive, siblings do not seem to offset these disadvantages in households with absent fathers. Their presence works in the opposite direction if looking at locus of control: the presence of siblings in the household is on average associated with more internal locus of control, and even though the interaction with father absence is also positive, it does not reach statistical significance, and therefore, the role of siblings cannot be said to be different between fatherless households and those with two parents.

## Robustness Checks

We have conducted a number of sensitivity analyses to test the robustness of our results. First, even if our substantive interest in this paper is about processes occurring at the individual level, and therefore the models shown throughout the paper are built relying on two levels (individual and country), we re-estimated our models considering an intermediate (i.e. school) level of analysis to appropriately account for the sampling methods in PISA and to address the potential correlation between family forms and school selection. Although the inclusion of the school level of analysis shrank the size of most coefficients of interest, the main conclusions of the paper remained unaltered. In other words, residential segregation and especially the concentration of students living without a father in particular schools seem to be part of the mechanisms explaining the disadvantages associated with certain household types. Second, results obtained using standard maximum likelihood approach to fit the multilevel structure instead of restricted maximum likelihood are substantively equivalent.

## Discussion and Conclusion

This paper has brought to the fore a number of relevant insights that improve our understanding of how household structure is associated with the educational outcomes of adolescent children in wealthy countries. To start with, we have shown that in line with the previous literature, there is a significant disadvantage associated with the absence of fathers in almost all the OECD countries analysed for the cognitive outcome analyzed here, i.e. scores in mathematics. Most of the research conducted so far is based on single-case studies and typically looks at either cognitive indicators or final educational attainment (see McLanahan et al. 2013 for a review). This paper adds to this literature by systematically studying international patterns of both cognitive and non-cognitive child outcomes associated with living in a household where the father is absent. On the one hand, we have documented existing country variations in the characteristic disadvantages of children’s fatherless households. On the other hand, we have demonstrated that the penalty associated with an absent father is larger for mathematics scores than it is for locus of control in all countries. In fact, the country-specific estimated effects of father absence on non-cognitive skills (locus of control) were often not statistically significant. This suggests that absent fathers seem to affect the educational opportunities of their offspring more through cognitive rather than non-cognitive mechanisms. This finding resonates with the idea developed in psychology that non-cognitive skills associated with personality traits tend to be more stable over the life course. Though not immune to the biographical shocks in the family domain that are often the cause of father absence (cf. Biblarz and Gottainer 2000), non-cognitive characteristics show, according to our findings, more inertia than cognitive ones.

Living in a multigenerational household that includes grandparents is broadly associated with a significant educational disadvantage in both cognitive and non-cognitive characteristics. In terms of numeracy, this disadvantage is found in almost all developed economies, which is consistent with previous evidence (Kreidl and Hubatková 2014). As suggested by previous research (de Lange et al. 2014; Ermisch and Härkönen in this issue), compositional effects are likely to play an important role here as adverse selection processes into multigenerational households including children and grandparents affect the scarcity of economic resources in those families. Although a harmful influence could also be detected for locus of control in most settings, the estimates do not tend to reach statistical significance. Further research, using more finely grained analyses, should aim to confirm whether the regularity we here identify stands considering selection issues.

Interestingly, we found great international variety in the way co-residence with siblings correlates with educational outcomes. However, the dominant trend in most countries consists of siblings correlating negatively with cognitive skills and positively with non-cognitive skills (albeit less consistent across countries). This is a noteworthy result since the specialized literature has mostly described living with siblings as an adverse circumstance due to dilution of resources in these households (Steelman et al. 2002; Sandefur et al. 2006). Yet, for the general case and in line with our expectations, brothers and sisters appear to enforce internal locus of control (widely believed to favour long-term attainment). This finding marries well with prior evidence emphasizing the beneficial effects of siblings on social skills (Downey and Condron 2004).

Finally, we have explored the interactions between the absence of fathers on the one hand and co-residence with siblings and grandparents on the other. The goal was to contribute to the pertinent literature, asking whether across OECD countries the absence of fathers might be mitigated by living with other family members. We found significant positive effects for the interaction between absent fathers and the presence of siblings in the household for cognitive outcomes. Although having siblings is generally negative for mathematics performance, this penalty is slightly smaller in fatherless households. However, living together with siblings does not offset the disadvantages associated with father absence. All in all, siblings seem to entail more benefits when the father is absent even if mathematics scores of children living in this family configuration (absent father, with siblings) are markedly lower than those in comparable households in which the father is present. In the case of non-cognitive outcomes, the presence of brothers or sisters is associated generally with more internal locus of control. Comparing households with and without a father, the presence of siblings does not seem to significantly alter the general pattern. Broadly in line with earlier research on emotional well-being (Ruiz and Silverstein 2007), the compensatory role of grandparents in fatherless households is relatively larger for locus of control than for numeracy.

We conclude by pointing out some of the limitations of our analysis. Data constraints led us to adopt a largely exploratory and descriptive approach to studying the association between family forms and educational outcomes. PISA’s strengths are the large number of cases and the international scale of the survey, as well as the availability of comparable quality measures of both cognitive and non-cognitive skills. Some of the studied family forms, such as co-residence with grandparents among fatherless households, are rather rare societal phenomena, making large sample size of paramount importance. However, the cross-sectional nature of the data prevents us from engaging in causal analysis or from systematically assessing the role of selection processes into different household arrangements. In addition, the nature of the data does not allow for exploring the reasons behind the absence of the father from the household, the duration of this family form or possible joint custody arrangements. Our indicator of father absence may not mean the same across countries depending on the prevalence of divorce and separation such that father absence does not exclude a significant involvement from fathers in parenting in some settings. The position of the interviewee in the sibship order as well as the ages of the siblings cannot be known either.

An open task for future research is to distil a clearer picture from the country comparisons contained in this study. Cross-national research on the influence of family forms on child well-being is an expanding field (Hampden-Thompson 2013; de Lange et al. 2014; Kreidl and Hubatková 2014; Bernardi and Radl 2014), but several issues with important policy implications remain insufficiently understood, including the role of contextual moderating factors that may explain outlier cases and idiosyncratic country-specific findings regarding the impact of single parenthood (Park 2007). Sometimes, the country-specific findings reported here varied across the two outcome measures even when considering the same household characteristic. Moreover, even when there seems to be a systematic pattern, there is rarely an obvious explanation. Future research should also attempt to further elucidate the international differences in the results for siblings discovered in this study as well as the macro-social characteristics responsible for them.

## Appendix

Tables 5, 6 and 7.

**Table 5:** Sample description

Country	ISO code	Father	Grandparents	Siblings	*N*
		Absent (%)	Present (%)	Present (%)	
Australia	AUS	12.4	8.2	90.8	12,847
Austria	AUT	12.6	25.5	84.4	4403
Belgium	BEL	11.2	4.4	88.4	7876
Canada	CAN	10.2	9.2	87.6	19,325
Switzerland	CHE	12.8	7.5	89.6	10,373
Chile	CHL	20.4	19.3	85.5	5897
Czech Republic	CZE	14.9	20.5	84.8	4994
Germany	DEU	11.9	18.2	83.5	3908
Denmark	DNK	14.4	2.9	85.1	6801
Spain	ESP	8.8	15.5	83.7	23,495
Estonia	EST	17.6	20.2	77.9	4210
Finland	FIN	15.0	2.7	76.9	7913
France	FRA	13.4	3.8	87.0	4169
Great Britain	GBR	14.5	5.7	88.2	11,203
Greece	GRC	7.6	19.5	86.6	4745
Hungary	HUN	17.8	12.6	80.7	4374
Ireland	IRL	9.9	8.1	93.2	4575
Iceland	ISL	9.3	4.0	88.4	3210
Italy	ITA	8.5	18.9	84.4	29,337
Japan	JPN	10.7	31.4	86.9	5918
Korea	KOR	6.8	18.4	88.6	4551
Luxembourg	LUX	10.8	8.4	88.4	4852
Mexico	MEX	13.7	26.1	91.8	26,836
Netherlands	NLD	10.2	2.0	90.7	4159
Norway	NOR	9.3	7.8	88.3	4270
New Zealand	NZL	17.5	6.1	83.2	3880
Poland	POL	15.1	23.3	77.9	4244
Portugal	PRT	11.0	23.4	80.9	5069
Slovak Republic	SVK	13.5	27.3	86.4	4155
Slovenia	SVN	9.7	39.8	85.8	5471
Sweden	SWE	7.8	4.4	89.8	4221
Turkey	TUR	3.9	21.0	93.3	4063
USA	USA	17.5	11.5	87.7	4308
Total		11.7	15.0	86.6	259,652

*Source* PISA 2012

**Table 6:** Factor analysis/correlation: locus of control scale

	Factor loading	Uniqueness
(1) One can succeed in mathematics with enough effort	0.71	0.50
(2) Doing well in mathematics is completely up to me	0.62	0.62
(3) If I wanted, I could perform well in mathematics	0.64	0.58
(4) I perform poorly in mathematics regardless of the effort I put in	−0.36	0.87

*Source* PISA 2012, own calculations. *N* = 33 countries

*Method* principal factors. Rotation: orthogonal varimax (Kaiser off)

**Table 7:** Country-level correlation matrix

	Estimated coefficient father absence on maths	Estimated coefficient father absence on LOC	Prevalence father absence	Estimated coefficient grandparents on maths	Estimated coefficient grandparents on LOC	Prevalence grandparents	Estimated coefficient siblings on maths	Estimated coefficient siblings on LOC	Incidence siblings
Father absence on maths (b)	1								
Father absence on LOC (b)	0.215	1							
Prevalence, father absence	−0.086	0.039	1						
Grandparents on maths (b)	0.220	0.088	0.211	1					
Grandparents on LOC (b)	−0.317	0.259	0.414	0.166	1				
Prevalence, Grandparents	0.394	0.334	−0.065	0.663	0.107	1			
Siblings on maths (b)	−0.314	−0.309	0.233	−0.058	0.229	−0.110	1		
Siblings on LOC (b)	0.069	−0.535	0.241	0.055	−0.067	0.015	0.411	1	
Prevalence, Siblings	−0.124	0.250	−0.498	−0.356	−0.096	−0.184	−0.207	−0.420	1

*Source* PISA 2012, own calculations. *N* = 33 countries

## Footnotes

1 Consistent with our strategy to handle missing data, in the models presented below each of the ten imputations uses one of the five plausible values provided by PISA. There were thus no missing data regarding numeracy in our original data set. Gender and father’s absence were also never missing. Neither was there missing information to speak of regarding age (0.03% missingness) or mother’s education (0.6%). Missingness was low for native born (1.5%) and siblings (4.1%) and higher for grandparents in the household (25.8%), as well as locus of control (34.8%).

2 A number of recent studies analyse systematically whether the impact of divorce differs according to parents’ socio-economic status (McLanahan and Percheski 2008; Bernardi and Radl 2014; Grätz 2015; Bernardi and Boertien 2016a, b).

## References

[CR2] Amato PR (2010). Research on divorce: Continuing trends and new developments. Journal of Marriage and Family.

[CR3] Amato PR, James S (2010). Divorce in Europe and the United States: Commonalities and differences across nations. Family Science.

[CR4] Antunes MJL, Ahlin EM (2014). Family management and youth violence: Are parents or community more salient?. Journal of Community Psychology.

[CR5] Astone NM, McLanahan SS (1991). Family structure, parental practices and high school completion. American Sociological Review.

[CR6] Au EWM (2015). Locus of control, self-efficacy, and the mediating effect of outcome control: Predicting course-level and global outcomes in an academic context. Anxiety, Stress, & Coping.

[CR7] Becker GS, Lewis HG (1973). On the interaction between the quantity and quality of children. Journal of Political Economy.

[CR8] Beechum NO (2012). Teaching adolescents to become learners. The role of noncognitive factors in shaping school performance: A critical literature review.

[CR9] Bernardi F (2014). Compensatory advantage as a mechanism of educational inequality: A regression discontinuity based in month of birth. Sociology of Education.

[CR10] Bernardi, F., & Boertien, D. (2016a). Understanding heterogeneity in the effects of parental separation on educational achievement in Britain: Do children from lower educational backgrounds have less to lose? *European Sociological Review,**32*(6), 807–819.

[CR11] Bernardi, F., & Boertien, D. (2016b). Non-intact families and diverging educational destinies: A decomposition analysis for Germany, Italy, the United Kingdom and the United States. *Social Science Research*. doi:10.1016/j.ssresearch.2016.09.004. 10.1016/j.ssresearch.2016.09.004 28202141

[CR12] Bernardi F, Radl J (2014). The long-term consequences of parental divorce for children’s educational attainment. Demographic Research.

[CR13] Biblarz TJ, Gottainer G (2000). Family structure and children’s success: A comparison of widowed and divorced single-mother families. Journal of Marriage and Family.

[CR14] Björklund A, Sundström M (2006). Parental separation and children’s educational attainment: A siblings analysis on Swedish register data. Economica.

[CR15] Bol T, Kalmijn M (2016). Grandparents’ resources and grandchildren’s schooling: Does grandparental involvement moderate the grandparent effect?. Social Science Research.

[CR16] Boudon R (1974). Education, opportunity, and social inequality: Changing prospects in western society.

[CR17] Breen R, Jonsson JO (2005). Inequality of opportunity in comparative perspective: Recent research on educational attainment and social mobility. Annual Review of Sociology.

[CR18] Castillo, M., Ferraro, P. J., Jordan, J. L., & Petrie, R. (2011). The today and tomorrow of kids: Time preferences and educational outcomes of children. *Journal of Public Economics,**95*(11–12), 1377–1385.

[CR19] Cavanagh SE, Huston AC (2008). The timing of family instability and children’s social development. Journal of Marriage and Family.

[CR20] Chase-Lansdale PL, Cherlin AJ, Kieman KE (1995). The long-term effects of parental divorce on the mental health of young adults: A developmental perspective. Child Development.

[CR21] Cherlin AJ, Chase-Lansdale PL, McRae C (1998). Effects of parental divorce on mental health throughout the life course. American Sociological Review.

[CR22] Cherlin AJ, Furstenberg FF, Chase-Lansdale PL, Kiernan KE, Robins PK, Morrison DR, Teitler JO (1991). Longitudinal studies of effects of divorce on children in Great Britain and the United States. Science.

[CR23] Chiu MM (2010). Effects of inequality, family and school on mathematics achievement: Country and student differences. Social Forces.

[CR24] Cobb-Clark DA, Schurer S (2013). Two economists’ musings on the stability of locus of control. The Economic Journal.

[CR25] Cooper H (1996). The effects of summer vacation on achievement test scores: A narrative and meta-analytic review. Review of Educational Research.

[CR26] de Lange M, Dronkers J, Wolbers M (2014). Single-parent family forms and children’s educational performance in a comparative perspective: Effects of school’s share of single-parent families. School Effectiveness and School Improvement.

[CR27] Downey DB (2001). Number of siblings and intellectual development. The resource dilution explanation. American Psychologist.

[CR28] Downey DB, Condron DJ (2004). Playing well with others in kindergarten: The benefit of siblings at home. Journal of Marriage and Family.

[CR29] Dronkers J (1999). The effects of parental conflicts and divorce on the well-being of pupils in Dutch secondary education. European Sociological Review.

[CR30] Dronkers J, Härkönen J (2008). The inter-generational transmission of divorce in cross-national perspective: Results from the fertility and family surveys. Population Studies.

[CR31] Duckworth AL, Peterson C, Matthews MD, Kelly DR (2007). Grit: Perseverance and passion for long-term goals. Journal of Personality and Social Psychology.

[CR32] Duckworth AL, Seligman MEP (2005). Self-discipline outdoes IQ in predicting academic performance of adolescents. Psychological Science.

[CR33] Erman, J., & Härkönen, J. (2017). Parental separation and school performance among children of immigrant mothers in Sweden. *European Journal of Population*. doi:10.1007/s10680-017-9419-3. 10.1007/s10680-017-9419-3 PMC5400781 28490831

[CR34] Ermisch J, Francesconi M (2001). Family matters: Impacts of family background on educational attainments. Economica.

[CR35] Ermisch J, Francesconi M, Pevalin J (2004). Parental partnership and joblessness in childhood and their influence on young people’s outcomes. Journal of the Royal Statistical Society Series A.

[CR36] Falbo T (1991). The impact of grandparents on children’s outcomes in China. Marriage and Family Review.

[CR37] Fraley C, Roberts BW (2005). Patterns of continuity: A dynamic model for conceptualizing the stability of individual differences in psychological constructs across the life course. Psychological Review.

[CR38] Gähler M, Palmtag EL (2015). Parental divorce, psychological well-being and educational attainment: Changed experience, unchanged effect among Swedes born 1892–1991. Social Indicators Research.

[CR39] Garber J, Seligman MEP (1980). Human helplessness: Theory and applications.

[CR40] Grätz M (2015). When growing up without a parent does not hurt: Parental separation and the compensatory effect of social origin. European Sociological Review.

[CR41] Gutman, L. M., & Schoon, I. (2013). The impact of non-cognitive skills on outcomes for young people. *Education Endowment Foundation*. http://educationendowmentfoundation.org.uk/uploads/pdf/Non-cognitive_skills_literature_review_1.pdf

[CR42] Hampden-Thompson G (2013). Family policy, family structure, and children’s educational achievement. Social Science Research.

[CR85] Hanushek, E. A., & Woessmann, L. (2012). Do better schools lead to more growth? Cognitive skills, economic outcomes, and causation. *Journal of Economic Growth, 17*(4), 267–321.

[CR43] Härkönen J (2014). Birth order effects on educational attainment and educational transitions in West Germany. European Sociological Review.

[CR44] Heckman JJ, Kautz T (2012). Hard evidence on soft skills. Labour Economics.

[CR45] House JD (2006). Mathematics beliefs and achievement of elementary school students in Japan and the United States: Results from the third international mathematics and science study. The Journal of Genetic Psychology.

[CR46] Hsin A (2012). Is biology destiny? Birth weight and differential parental treatment. Demography.

[CR47] Jæger MM (2012). The extended family and children’s educational success. American Sociological Review.

[CR48] Jonsson JO, Gähler M (1997). Family dissolution, family reconstitution, and children’s educational careers: Recent evidence for Sweden. Demography.

[CR49] Kalmijn M, van de Werfhorst HG (2016). Sibship size and gendered resource dilution in different societal contexts. PLoS ONE.

[CR50] Kao G, Tienda M (1998). Educational aspirations of minority youth. American Journal of Education.

[CR51] Keith, V. M., & Finlay, B. (1988). The impact of parental divorce on children’s educational attainment, marital timing, and likelihood of divorce. *Journal of Marriage and the Family,**50*(3), 797–809.

[CR52] Kim HS (2011). Consequences of parental divorce for child development. American Sociological Review.

[CR53] Kim LS, Sandler IN, Tein JY (1997). Locus of control as a stress moderator and mediator in children of divorce. Journal of Abnormal Child Psychology.

[CR54] Kreidl M, Hubatková B (2014). Does co-residence with grandparents reduce the negative association between sibship size and reading test scores? Evidence from 40 countries. Research in Social Stratification and Mobility.

[CR55] Lawson DW, Mace R (2010). Siblings and childhood mental health: Evidence for a later-born advantage. Social Science and Medicine.

[CR56] Lawson DW, Makoli A, Goodman A (2013). Sibling configuration predicts individual and descendant socioeconomic success in a modern post-industrial society. PLoS ONE.

[CR57] McLanahan S (2004). Diverging destinies: How children are faring under the second demographic transition. Demography.

[CR86] McLanahan, S., & Percheski, C. (2008). Family structure and the reproduction of inequalities. *Annual Review of Sociology, 34*(1), 257–276.

[CR58] McLanahan S, Tach L, Schneider D (2013). The causal effects of father absence. Annual Review of Sociology.

[CR59] Meier LL, Semmer NK, Elfering A, Jacobshagen N (2008). The double meaning of control: Three-way interactions between internal resources, job control, and stressors at work. Journal of Occupational Health Psychology.

[CR60] Modin B, Fritzell J (2009). The long arm of the family: Are parental and grandparental earnings related to young men’s body mass index and cognitive ability?. International Journal of Epidemiology.

[CR61] Møllegaard S, Jæger MM (2015). The effect of grandparents’ economic, cultural, and social capital on grandchildren’s educational success. Research in Social Stratification and Mobility.

[CR63] Ní Bhrolcháin M (2001). ‘Divorce effects’ and causality in the social sciences. European Sociological Review.

[CR64] O’Connell M, Sheikh H (2008). Achievement-related attitudes and the fate of ‘At-Risk’ groups in society. Journal of Economic Psychology.

[CR65] OECD. (2014). *PISA 2012.* Technical report. Paris: OECD.

[CR66] OECD. (2015a). *Does math make you anxious? PISA in Focus no. 48*. Paris: OECD.

[CR67] OECD. (2015b). *How confident are students in their ability to solve mathematics problems? PISA in Focus no. 56*. Paris: OECD.

[CR68] Park H (2007). Single parenthood and children’s reading performance in Asia. Journal of Marriage and Family.

[CR69] Pong SL, Ju DB (2000). The effects of change in family structure and income on dropping out of middle and high school. Journal of Family Issues.

[CR70] Rotter JB (1975). Some problems and misconceptions related to the construct of internal versus external control of reinforcement. Journal of Consulting and Clinical Psychology.

[CR71] Ruiz SA, Silverstein M (2007). Relationships with grandparents and the emotional well-being of late adolescent and young adult grandchildren. Journal of Social Issues.

[CR72] Sandberg JF, Hofferth SL (2001). Changes in parental time with children. Demography.

[CR73] Sandefur G, Meier A, Campbell ME (2006). Family resources, social capital, and college attendance. Social Science Research.

[CR74] Scott, M. E., DeRose, L. F., Lippman, L. H., & Cook, E. (2013). Two, one, or no parents? Children’s living arrangements and educational outcomes around the world. In Child Trends (Ed.). *World family map 2013. Mapping family change and child well*-*being outcome*s (pp. 48–66). An international report, Washington, DC.

[CR75] Shirahase S, Raymo JM (2014). Single mothers and poverty in Japan: The role of intergenerational coresidence. Social Forces.

[CR76] Sigle-Rushton W, Lyngstad TH, Andersen PL, Kravdal Ø (2014). Proceed with caution? Parents’ union dissolution and children’s educational achievement. Journal of Marriage and Family.

[CR77] Steelman LC, Powell B, Werum R, Carter S (2002). Reconsidering the effects of sibling configuration: Recent advances and challenges. Annual Review of Sociology.

[CR78] Sullivan, A., Ketende, S., & Joshi, H. (2013). Social class and inequalities in early cognitive scores. *Sociology, 47*(6), 1187–1206.

[CR79] Sun Y, Li Y (2002). Children’s well-being during parents’ marital disruption process: A pooled time-series analysis. Journal of Marriage and Family.

[CR80] Tiruchittampalam, S., Nicholson, T., Levin, J. R., & Ferron, J. M. (2016). The effects of preliteracy knowledge, schooling, and summer vacation on literacy acquisition. *The Journal of Educational Research*. doi:10.1080/00220671.2016.1190911.

[CR81] Uphold-Carrier H, Utz R (2012). Parental divorce among young and adult children: A long-term quantitative analysis of mental health and family solidarity. Journal of Divorce & Remarriage.

[CR82] Wu Q (2014). Motivations and decision-making processes of Mainland Chinese students for undertaking master’s programs abroad. Journal of Studies in International Education.

[CR83] Zau, A., & Betts, J. R. (2008). *Predicting success, preventing failure: An investigation of the California high school exit exam*. Public Policy Instit. of CA.

[CR84] Zeng Z, Xie Y (2014). The Effects of grandparents on children’s schooling: Evidence from rural China. Demography.

